# Knowledge, Attitudes, and Uptake of Cervical Cancer Screening Among Female Students and Staff at a Tertiary Institution in Northern Uganda: A Cross‐Sectional Study

**DOI:** 10.1002/hsr2.73191

**Published:** 2026-09-02

**Authors:** Milton Anguyo, Morrish Obol Okello, David Komakec, Emmanuel Alyoomu, Habert Aziku, Ruth Catherine Ouma, Pebalo Francis Pebolo

**Affiliations:** ^1^ Faculty of Medicine Gulu University Gulu Uganda; ^2^ Faculty of Pharmacy Eastern Mediterranean University Famagusta Cyprus; ^3^ Department of Obstetrics and Gynaecology Faculty of Medicine Gulu University Gulu Uganda

**Keywords:** attitude, cervical cancer, females, knowledge, Northern Uganda, screening, tertiary institution

## Abstract

**Background and Aims:**

Cervical cancer remains a leading cause of cancer‐related deaths among women in low‐resource settings like Uganda. Screening is essential for early detection and prevention, yet knowledge, attitudes, and uptake among university females are poorly understood. This study aimed to investigate knowledge, attitudes, and uptake of cervical cancer screening among females at a tertiary institution in Northern Uganda.

**Methods:**

A cross‐sectional quantitative study was conducted at a tertiary institution in Northern Uganda, involving 335 females aged 25–49 years (students and staff) selected via stratified random sampling (from a calculated target of 336; one questionnaire was excluded for incompleteness). Data were collected using a structured questionnaire and analyzed with Stata version 17 (StataCorp, College Station, TX, USA). Descriptive statistics summarized knowledge, attitudes, and uptake. Logistic regression examined factors associated with screening uptake. All statistical tests were two‐sided, and a *p* < 0.05 was considered statistically significant.

**Results:**

A total of 335 women (median age 27 years, IQR 26–30) were analyzed. Awareness of cervical cancer was almost universal (97.9%, 328/335), with health institutions the commonest information source (50.0%, 164/328). Overall, 83.3% (279/335) demonstrated good knowledge, although knowledge of specific items such as HPV and screening methods was substantially lower. Attitudes were mixed: 52.5% (176/335) negative and 47.5% (159/335) positive towards screening. Uptake of cervical cancer screening was 31.3% (105/335). Among the unscreened (68.7%, 230/335), barriers included lack of screening‐location information (84/230, 35.4%), pain concerns (78/230, 32.9%), perceived good health (56/230, 23.6%), and shyness (19/230, 8.0%). In multivariable logistic regression, older age (aOR 1.11, 95% CI 1.05–1.18, *p* < 0.001) and being staff (aOR 2.37, 95% CI 1.15–4.92, *p* = 0.02) were significantly associated with higher screening uptake.

**Conclusion:**

Despite high overall knowledge, more than half of participants demonstrated negative attitudes toward screening, and uptake remained low, revealing a substantial knowledge–attitude‐practice gap. Targeted education to University females and improved service access interventions are recommended to enhance screening and reduce cervical cancer burden.

## 1 Introduction

Cervical cancer remains a major global health challenge, ranking as the fourth most common cancer among women worldwide [1]. In 2022, approximately 660,000 new cases were diagnosed globally, resulting in around 350,000 deaths, with the vast majority occurring in low‐ and middle‐income countries (LMICs) where access to preventive services is limited [1]. The disease is primarily caused by persistent infection with high‐risk human papillomavirus (HPV) genotypes, such as HPV‐16 and HPV‐18, which are transmitted sexually and account for over 70% of cases [2]. Key risk factors include early onset of sexual activity, multiple sexual partners, high parity, prolonged oral contraceptive use, and co‐infection with HIV, which exacerbates susceptibility due to immune suppression [3, 4, 5]. Despite being largely preventable through vaccination, screening, and early treatment, cervical cancer continues to impose significant morbidity, mortality, and economic burdens, particularly in resource‐constrained settings where late‐stage diagnoses predominate [6].

Sub‐Saharan Africa bears a disproportionate impact of cervical cancer, with one of the highest burdens due to intersecting factors such as high HIV prevalence, limited healthcare infrastructure, and sociocultural barriers [7, 8]. In this region, cervical cancer is a leading cause of cancer‐related deaths among women, with incidence rates often exceeding 30 cases per 100,000 women annually [9, 10]. Uganda equally suffers from these challenges, reporting an estimated 6413 new cases and over 4000 deaths yearly, making it the leading cause of cancer mortality among women [11]. Compounding this, the COVID‐19 pandemic diverted resources from non‐communicable disease programs, including cervical cancer screening, leading to further declines in uptake in low‐resource [12]. Structural barriers, such as inadequate screening facilities, financial constraints, and transportation issues, alongside individual factors like fear of pain, stigma, and low perceived risk, perpetuate low screening rates, often below 30% in rural populations [13, 14, 15].

Effective cervical cancer control relies on early detection through screening methods like visual inspection with acetic acid (VIA), Pap smears, or HPV testing, which can reduce mortality by up to 90% when implemented routinely [1]. However, uptake remains suboptimal across sub‐Saharan Africa, with a pooled prevalence of 12.87%, influenced by low knowledge, negative attitudes, HIV disease, and barriers such as cost and accessibility [16]. In Uganda, national guidelines recommend annual screening for HIV‐positive women and every 3 years for others, yet overall uptake lies around 20%–40% in general populations, with even lower rates in rural districts [17, 18]. Studies highlight persistent knowledge gaps regarding HPV as the causative agent and symptoms like abnormal bleeding, alongside attitudinal barriers including misconceptions about screening procedures [19, 20].

University populations include young, educated women who may face elevated risk through behavioral and structural factors, such as early or multiple sexual partnerships and delayed initiation of screening, and who are therefore an important group for targeted preventive interventions [12, 21]. Research in Uganda and neighboring countries reveals moderate awareness but significant deficits in specific knowledge and positive attitudes toward screening among students. For instance, in Western Uganda, only 39% of university students demonstrated good knowledge, with uptake at 20.3%, linked to non‐medical fields of study and low risk perception [12]. In Ethiopia, undergraduate students showed limited understanding of screening practices, with barriers including embarrassment and lack of information [21]. In Northern Uganda, where cervical cancer incidence is amplified by post‐conflict recovery challenges and high‐risk behaviors in non‐residential university settings, data on knowledge, attitudes, and uptake among tertiary institution females are scarce. Gaps in specific knowledge about causative agents and barriers like pain concerns or shyness may hinder uptake, despite general awareness.

Taken together, these findings carry direct implications for the present study. The consistently reported pattern of high awareness alongside low screening uptake indicates that awareness alone is insufficient to drive preventive behavior, so interventions must address knowledge of specific screening methods, attitudes, and structural access rather than general awareness. In Uganda, where cervical cancer is the leading cause of cancer‐related death among women and most cases present at advanced, less treatable stages, knowledge, attitudes, and uptake (KAP) sit at the center of early detection: adequate knowledge enables women to recognize risk and the value of screening, favorable attitudes translate that knowledge into intention, and actual uptake of Pap smear, VIA, or HPV testing is the behavior that permits detection of precancerous lesions before progression. Understanding how these three domains interact in an educated but under‐screened population, therefore, situates the research problem, namely, why a group with high awareness still achieves low screening coverage, and identifies the specific leverage points (specific knowledge, attitude change, and on‐site access) through which uptake and, ultimately, early detection can be improved in this setting.

Unlike previous studies that focused exclusively on students, this study uniquely included both female students and staff within a non‐residential, rural, post‐conflict university setting. This dual inclusion provides nuanced insights into how age, institutional role (student vs. staff), and positioning within the academic environment differentially shape cervical cancer screening behaviors, knowledge translation, and access to preventive services. By examining these factors together, the study addresses an important evidence gap and offers more comprehensive evidence to inform institution‐wide interventions that can bridge the uptake divide between these two key subgroups. This study, therefore, aimed to determine the knowledge, attitudes, uptake, and associated factors among female students and staff at a tertiary institution in Northern Uganda.

## 2 Methods

### 2.1 Study Design

This was an institution‐based cross‐sectional study employing a quantitative research design conducted among female students and staff at a tertiary institution in Northern Uganda from April 2, 2025, to June 14, 2025.

### 2.2 Study Setting

The study was conducted at Gulu University, a public tertiary institution located in Northern Uganda, approximately 333 km north of Kampala, the capital city. The institution comprises six faculties (Agriculture and Environment, Medicine, Business and Development Studies, Law, Science, and Education) offering over 80 academic programs. It is a non‐residential university, with most female students residing in privately owned hostels or rentals off‐campus, while staff reside in their respective homes.

At the time of the study, the university had approximately 4200 female students distributed across the six faculties and about 350 female staff. The approximate female student distribution by faculty was Agriculture and Environment (about 520), Medicine (about 610), Business and Development Studies (about 1150), Law (about 430), Science (about 690), and Education (about 800). These faculty‐level and staff population figures formed the basis for the probability‐proportional‐to‐size (Neyman) allocation of the calculated sample across the strata, so that each faculty and the staff group contributed in proportion to its size (exact institutional figures were obtained from the University Academic Registrar and Human Resource records).

### 2.3 Study Population and Eligibility

The study population consisted of female students and staff aged 25–49 years at Gulu University. This age range was chosen because it corresponds to the ages of peak incidence of invasive cervical cancer in Uganda and to the WHO‐recommended target ages for routine cervical cancer screening (screening prioritized at 35 and 45 years), making these women the group for whom screening uptake is most clinically relevant; younger undergraduates below 25 years were therefore not the focus, a decision revisited in the limitations.

### 2.4 Inclusion and Exclusion Criteria

Inclusion criteria:

- Female students currently enrolled across the faculties at the main campus, or female staff of the university.
- Aged 25–49 years at the time of data collection.
- Willing and able to provide written informed consent.

Exclusion criteria:

- Women with severe illness that impaired their ability to give informed consent or complete the questionnaire.
- Eligible women who were absent from campus during the data‐collection period.

### 2.5 Sample Size Estimation

The sample size was calculated using the Kish and Leslie (1965) formula for an infinite population [22]:

$n=\frac{Z^{2}\mathrm{pq}}{d^{2}}$, where n = required sample size, *Z* = 1.96 (standard normal deviate at the 95% confidence level), *p* = 0.677 estimated proportion of women with awareness of cervical cancer screening, based on Getaneh et al., 2021, and d = margin of error (set at 0.05), with *q* = 1 − *p* = 0.323.

$n=\frac{1.96^{2}\times 0.677\times 0.323}{0.05^{2}},$ *n* = 336 participants.

The calculation drew on an estimated source population of approximately 4550 eligible women aged 25–49 years (about 4200 female students and about 350 female staff in this age band). The 25–49‐year category was retained because it reflects the ages of highest cervical cancer risk and the WHO‐recommended screening ages, as noted above. The resulting sample of 336 was distributed across the six faculties and the staff stratum using proportionate stratified sampling, so that each stratum contributed a number of participants proportional to its share of the eligible female population (probability proportional to size); within each stratum, participants were then selected at random as described below. True Neyman allocation was not applied because stratum‐specific variances were not available a priori.

### 2.6 Sampling Procedure

Stratified random sampling was employed to ensure representation across faculties and roles (students and staff). The population was stratified by faculty for students and by role (staff vs. students). The total sample of 336 was allocated to each faculty (for students) and to the staff stratum by proportionate stratified sampling (probability proportional to size), so that larger faculties contributed proportionally more participants. Within each stratum, a sampling frame of eligible females was compiled from faculty class registers and the staff nominal roll, and participants were selected by computer‐generated random numbers (simple random sampling within strata). The women whose identifiers were drawn were then traced and invited individually: class representatives forwarded the study invitation to the specific selected students through faculty WhatsApp groups and read out their names during class announcements, while selected staff were approached directly at their offices. Thus, WhatsApp groups and class announcements were used only as channels to reach already‐randomly‐selected individuals, not as an open call for volunteers; nonetheless, because participation remained voluntary, some self‐selection cannot be excluded, and this is addressed in the limitations. Informed consent was obtained prior to participation.

### 2.7 Data Collection Tool and Procedure

A structured, self‐administered questionnaire was adapted from a validated instrument developed by Getaneh et al. [21], which assessed knowledge, attitudes, and practices toward cervical cancer screening among university female students in Ethiopia. The tool was refined for cultural and contextual relevance to the Northern Ugandan setting through expert review by two obstetrician‐gynecologists and a public‐health researcher. It was pilot‐tested on 20 eligible females (excluded from the final analysis), after which minor wording changes were made. Internal consistency was acceptable, with Cronbach's alpha of 0.78 for the knowledge items and 0.74 for the attitude items. The questionnaire included sections on sociodemographic characteristics (age, religion, role, year of study, and faculty), KAP of cervical cancer screening.

Knowledge was assessed through questions on awareness of cervical cancer, causative agent (HPV), symptoms, risk factors, prevention methods, treatment options, screening methods (e.g., Pap smear and VIA; note that biopsy, though listed as a response option, is a diagnostic rather than a screening procedure), and recommended screening frequency. Each correct response was scored 1 point, with incorrect or “don't know” responses scored 0. Knowledge was assessed with 8 composite items covering awareness, causative agent, symptoms, risk factors, prevention, treatment, target group, and screening methods, giving a maximum possible score of 8. The total score was converted to a percentage, and participants scoring ≥ 60% were categorized as having good knowledge, consistent with the ≥ 60% (Bloom's modified) cutoff widely used in comparable knowledge‐attitude‐practice studies; those below 60% were classified as having poor knowledge. Attitudes were measured using 9 Likert‐scale items (strongly agree = 5 to strongly disagree = 1) assessing perceptions of personal risk, seriousness of cervical cancer, benefits of early detection and screening, curability, and willingness to be screened. Responses were summed to obtain a total attitude score (range 9–45); no items were reverse‐scored because all nine items were positively worded. As the summed attitude score was approximately symmetrically distributed, the sample mean was used as the classification threshold: participants scoring ≥ the mean were classified as having a more favorable attitude, and those below the mean as having a less favorable attitude. We acknowledge that a sample‐mean split is a relative classification and does not establish an absolute positive or negative attitude; the continuous attitude score and item‐level responses are therefore also presented. Uptake of cervical cancer screening was defined as a self‐reported history of ever having undergone any recognized screening method (Pap smear, VIA, or HPV testing) at any time in the past (“ever screened,” yes/no). Pap‐smear experience was recorded separately, and reasons for non‐uptake were collected to contextualize barriers.

The questionnaire was available in English, the medium of instruction at the institution was paper‐based. Data were collected using self‐administered questionnaires distributed physically. Research assistants explained the study purpose, voluntary nature, and confidentiality in private settings. Questionnaires were anonymous, with unique codes for tracking.

### 2.8 Data Management

Completed questionnaires were checked for completeness. Data were entered into a password‐protected Excel spreadsheet and exported to Stata version 17 (StataCorp, College Station, TX, USA) for further analysis. Double‐entry verification was performed for 10% of records to minimize errors.

### 2.9 Data Analysis

Data were cleaned for inconsistencies and missing values. At Descriptive statistics numerical variable age was reported as median and interquartile range, because of its non‐normality. Normality was tested using the Shapiro–Wilk test. Frequencies and percentages were used to summarize sociodemographic characteristics, KAP, as categorical variables at univariable analysis. Variables associated with uptake at *p* < 0.20 in bivariable (unadjusted) logistic regression, together with the a priori confounders age and institutional role, were entered into a multivariable logistic regression model to identify factors independently associated with screening uptake. Knowledge and attitude were also tested in the adjusted model; they were retained or dropped on the basis of the *p* < 0.20 screening criterion and are reported accordingly. Collinearity between age and staff/student status was assessed using variance inflation factors (all < 2). Model fit was evaluated with the Hosmer–Lemeshow goodness‐of‐fit test. Crude and adjusted odds ratios (cOR, aOR) with 95% confidence intervals and exact p‐values are reported. All tests were two‐sided, and a *p* < 0.05 was considered statistically significant. Analyses were conducted in Stata version 17 (StataCorp, College Station, TX, USA); these were prespecified analyses except where noted as exploratory.

## 3 Results

### 3.1 General Characteristics of the Female Students and Staff of Gulu University

Note: Percentages for Year of Study and Faculty are calculated among students (*n* = 279); percentages for the Staff category (Administration, Support staff) are calculated among staff (*n* = 56). The Staff/Student row percentages are of the total sample (*n* = 335). Rows marked “if student” exclude the 56 staff members from the denominator.

A total of 335 participants completed the study (response rate: 99.7%), all of whom fully completed the self‐administered questionnaire. The median age was 27 years (IQR: 26–30). Regarding religious affiliation, Catholic was dominant (*n* = 146, 43.6%). In terms of role, 16.7% (*n* = 56) were staff members and 83.3% (*n* = 279) were students.

Among the students (*n* = 279), 26.2% (*n* = 73) were in Year 1, 40.1% (*n* = 112) in Year 2, 24.7% (*n* = 69) in Year 3, 8.6% (*n* = 24) in Year 4, and 0.4% (*n* = 1) in Year 5. Faculty distribution among students showed the highest representation from the Faculty of Education (*n* = 103, 30.8%), followed by Faculty of Business (*n* = 80, 23.9%), Faculty of Agriculture (*n* = 38, 11.3%), support staff (*n* = 30, 9.0%), Administration (*n* = 26, 7.8%), Faculty of Law (*n* = 26, 7.8%), Faculty of Medicine (*n* = 24, 7.2%), and Faculty of Science (*n* = 8, 2.4%). Table 1 presents a detailed summary of the sociodemographic characteristics of the participants.

**Table 1:** Social and demographic characteristics among female university students and staff of Gulu University.

Variable	Frequency	Percentage (%)
***N***	335	100.0
**Age in years**		
Median (IQR)	27	26–30
**Religious affiliation**		
Anglican	109	32.5
Born Again	55	16.4
Catholic	146	43.6
Other	25	7.5
**Staff/Student**		
Staff	56	16.7
Student	279	83.3
**Year of study (if student)**		
Year 1	73	26.2
Year 2	112	40.1
Year 3	69	24.7
Year 4	24	8.6
Year 5	1	0.4
**Department/Major (if student)**		
Administration	26	7.8
Faculty of Agriculture	38	11.3
Faculty of Business	80	23.9
Faculty of Education	103	30.8
Faculty of Law	26	7.8
Faculty of Medicine	24	7.2
Faculty of Science	8	2.4
Support staff	30	9.0

Abbreviation: IQR, interquartile range.

### 3.2 Knowledge of Cervical Cancer Screening Among Female University Students and Staff of Gulu University

Nearly all participants (*n* = 328, 97.9%) had heard about cervical cancer, with health institutions being the primary source of information (*n* = 164, 50.0% of those aware), followed by news media (*n* = 93, 28.4%), teachers (*n* = 54, 16.5%), family/friends/neighbors (*n* = 35, 10.7%), and magazines (*n* = 6, 1.8%).

Regarding the causative agent, less than half correctly identified a virus (*n* = 150, 44.8%), while 37.0% (*n* = 124) did not know, and incorrect responses included bacteria (*n* = 53, 15.8%), fungi (*n* = 7, 2.1%), and parasites (*n* = 1, 0.3%). For symptoms of cervical cancer, 35.5% (*n* = 119) correctly identified all three listed symptoms (vaginal foul‐smelling discharge, irregular vaginal bleeding, and post‐coital bleeding), while 29.6% (*n* = 99) did not know any symptoms.

Knowledge of risk factors varied: 29.0% (*n* = 97) identified multiple sexual partners, 22.7% (*n* = 76) identified HPV, 6.3% (*n* = 21) early sexual intercourse, and 2.7% (*n* = 9) cigarette smoking. Only 19.7% (*n* = 66) correctly identified all four risk factors, while an equal proportion (*n* = 66, 19.7%) did not know any. On prevention, 30.8% (*n* = 103) recognized HPV vaccination, 24.2% (*n* = 81) avoided multiple sexual partners, 4.8% (*n* = 16) avoided early sexual intercourse, and 2.7% (*n* = 9) quit smoking. A notable 24.2% (*n* = 81) correctly identified all four prevention mechanisms, but 13.4% (*n* = 45) did not know any preventive measures.

A large majority believed cervical cancer is curable if detected early (*n* = 283, 84.5%), while 12.2% (*n* = 41) were unsure, and 3.3% (*n* = 11) believed it was not. For treatment options, 25.7% (*n* = 86) correctly identified all three (surgery, specific drugs, radiotherapy), with individual mentions of specific drugs (*n* = 87, 26.0%), surgery (*n* = 51, 15.2%), and radiotherapy (*n* = 44, 13.1%); 20.0% (*n* = 67) did not know any treatment options.

Regarding screening, 52.8% (*n* = 177) correctly identified women over 25 years as the target group, and 40.3% (*n* = 135) recognized that all women should be screened. For recommended screening frequency, 41.5% (*n* = 139) believed annual screening, 27.2% (*n* = 91) every 3 years, and 20.6% (*n* = 69) were unsure. Knowledge of screening methods was limited: 44.2% (*n* = 148) knew none, 19.7% (*n* = 66) knew Pap smear, 12.8% (*n* = 43) VIA, 11.3% (*n* = 38) biopsy, and 11.9% (*n* = 40) all methods. Overall, 83.3% (*n* = 279) demonstrated good knowledge of cervical cancer and screening, while 16.7% (*n* = 56) had poor knowledge. Detailed results are presented in Table 2.

**Table 2:** Knowledge of cervical cancer screening among female university students and staff of Gulu University.

Variable	Frequency	Percentage (%)
***N***	335	100.0
**Have you ever heard about cervical cancer?**		
Yes	328	97.9
No	7	2.1
**Where did you learn about cervical cancer? (*n*** = **328)**		
Teachers	54	16.5
News media	93	28.4
Health institutions	164	50.0
Family, friends, neighbors	35	10.7
Magazine	6	1.8
**What is the causative agent of cervical cancer?**		
Virus	150	44.8
Bacteria	53	15.8
Fungi	7	2.1
Parasite	1	0.3
Don't know	124	37.0
**What are the symptoms of carcinoma of the cervix?**		
Vaginal foul‐smelling discharge	41	12.2
Vaginal irregular bleeding	49	14.6
Post coital bleeding	27	8.1
All of the three symptoms	119	35.5
Don't know	99	29.6
**Do you know the risk factors for cancer of the cervix?**		
Having multiple sexual partners	97	29.0
Human papillomavirus	76	22.7
Early sexual intercourse	21	6.3
Cigarette smoking	9	2.7
All four risks	66	19.7
Don't know	66	19.7
**How can a person prevent getting cancer of the cervix?**		
Avoid multiple sexual partners	81	24.2
Avoid early sexual intercourse	16	4.8
HPV vaccination	103	30.8
Quit cigarette smoking	9	2.7
All four prevention mechanisms	81	24.2
Don't know	45	13.4
**Can cancer of the cervix be cured in its earliest stages?**		
Yes	283	84.5
No	11	3.3
Don't know	41	12.2
**Variable**	**Frequency**	**Percentage (%)**
**How can someone with cancer of the cervix be treated?**		
Surgery	51	15.2
Specific drugs are given by a hospital	87	26.0
Radiotherapy	44	13.1
All	86	25.7
Don't know	67	20.0
**Screening frequency**		
Once a year	139	41.5
Every 3 years	91	27.2
Every 5 years	17	5.1
All	19	5.7
Don't know	69	20.6
**Who should be screened?**		
Women of > 25 years	177	52.8
Prostitutes	2	0.6
Elderly women	3	0.9
All	135	40.3
Don't know	18	5.4
**Which screening method do you know?**		
Pap smear	66	19.7
Biopsy^a^	38	11.3
Visual inspection with acetic acid	43	12.8
All	40	11.9
Don't know	148	44.2
**Overall knowledge**		
Good Knowledge	279	83.3
Poor Knowledge	56	16.7

^a^ Biopsy is a diagnostic procedure, not a screening method. Its inclusion as a response option assessed participants' ability to distinguish screening from diagnostic tests; the 11.3% selecting biopsy are interpreted as reflecting a knowledge gap rather than correct screening knowledge.

### 3.3 Attitudes of Female Students and Staff of Gulu University Towards Cervical Cancer Screening

The majority (78.4%, 262/335) of participants strongly agreed that it is helpful to detect cervical cancer early. A significant portion (39.1%, *n* = 131) believed they had a chance of getting cervical cancer, with 22.1% (*n* = 74) strongly agreeing with this statement. Regarding the seriousness of getting cervical cancer, 35.8% (*n* = 120) strongly believed it was serious for them, with another 28.7% (*n* = 96) agreeing. A majority (47.5%, *n* = 159) believed there are effective methods to reduce the risk and seriousness of cervical cancer, with 43.6% (*n* = 146) agreeing.

A significant majority (57.3%, *n* = 192) strongly agreed that carcinoma of the cervix is the cause of death, and 40.6% (*n* = 136) strongly believed that any woman could acquire cervical cancer, with another 34.9% (*n* = 117) agreeing. A considerable portion (43.0%, *n* = 144) believed that carcinoma of the cervix can be treated, with 37.0% (*n* = 124) strongly agreeing. Additionally, 37.3% (*n* = 125) strongly believed that screening helps in the prevention of cervical cancer, with 36.7% (*n* = 123) agreeing. When it came to willingness for screening, 43.6% (*n* = 146) strongly agreed, and 35.8% (*n* = 120) agreed that they were willing to be screened. Overall, 52.5% (*n* = 176) of the participants demonstrated a negative attitude towards cervical cancer screening, while 47.5% (*n* = 159) had a positive attitude. Details in Table 3.

**Table 3:** Attitudes of female students and staff of Gulu University towards cervical screening.

Variable (*N* = 335)	SA (*n*, %)	A (*n*, %)	N (*n*, %)	D (*n*, %)	SD (*n*, %)
Do you think it is helpful to detect cervical cancer early?	262 (78.4)	60 (18.0)	7 (2.1)	3 (0.9)	2 (0.6)
Do you believe that you have the chance of getting Cervical Cancer?	74 (22.1)	131 (39.1)	59 (17.6)	36 (10.8)	35 (10.4)
Do you believe that getting Cervical Cancer is serious for you?	120 (35.8)	96 (28.7)	59 (17.6)	40 (11.9)	20 (6.0)
Do you think that there are effective methods to reduce the risk of cervical cancer?	159 (47.5)	146 (43.6)	23 (6.9)	5 (1.5)	2 (0.6)
Do you think Carcinoma of the cervix is the cause of death?	192 (57.3)	84 (25.1)	46 (13.7)	9 (2.7)	4 (1.2)
Do you think any woman can acquire cervical cancer?	136 (40.6)	117 (34.9)	39 (11.6)	39 (11.6)	4 (1.2)
Do you think carcinoma of the cervix can be treated?	124 (37.0)	144 (43.0)	51 (15.2)	10 (3.0)	6 (1.8)
Do you think screening helps in the prevention of cervical cancer?	125 (37.3)	123 (36.7)	47 (14.0)	32 (9.6)	8 (2.4)
Willingness for screening	120 (35.8)	146 (43.6)	48 (14.3)	17 (5.1)	4 (1.2)

Abbreviations: A, agree; D, disagree; N, neutral; SA, strongly agree; SD, strongly disagree.

### 3.4 Uptake of Cervical Cancer Screening by the Female Students and Staff of Gulu University

The uptake of cervical cancer screening was 31.3%, with 105 participants reporting having been screened. Among those who had not been screened (*n* = 230), the reasons included not being informed about screening places (84/230, 35.4%), concerns about pain (78/230, 32.9%), believing they were healthy (56/230, 23.6%), and feeling shy (19/230, 8.0%). In terms of Pap smear tests, 82.7% (*n* = 277) of participants had never had one, while 17.3% (*n* = 58) had undergone the procedure. The reasons given for not having a Pap smear (*n* = 277) included never having heard of it (69.3%, *n* = 192), lack of interest (19.5%, *n* = 54), lack of time (9.0%, *n* = 25), and partners not allowing it (2.2%, *n* = 6). Table 4 provides more details.

**Table 4:** Uptake of cervical cancer screening by the female students and staff of Gulu University.

Variable	Frequency	Percentage (%)
**Have you ever been screened for cervical cancer?**		
Yes	105	31.3
No	230	68.7
**If not yet, what is the reason for not screening? (*n*** = **230)**		
I am healthy	56/230	23.6
It may be painful	78/230	32.9
I feel shy	19/230	8.0
I'm not informed about the screening place	84/230	35.4
**Have you had a Pap smear done before?**		
Yes	58	17.3
No	277	82.7
**If not done, why? (*n*** = **277)**		
No interest	54	19.5
Partners will not allow	6	2.2
No time	25	9.0
Never heard of it	192	69.3

### 3.5 Factors Associated With Uptake of Cervical Cancer Screening Among Female Students and Staff of Gulu University

In bivariable analysis, each additional year of age increased the odds of screening by 15%. Being staff (compared to students) was strongly associated with uptake (cOR 4.98, 95% CI: 2.72–9.11, *p* < 0.001). Compared to participants who identified as Anglican (reference group), those who were Born Again had similar odds of screening uptake (crude OR 1.07, 95% CI: 0.54–2.15, *p* = 0.842), as did those who were Catholic (crude OR 1.05, 95% CI: 0.61–1.79, *p* = 0.865). Participants belonging to other religions had lower but non‐significant odds of uptake (crude OR 0.70, 95% CI: 0.26–1.90, *p* = 0.480). Being staff (compared to students) was strongly associated with higher screening uptake (crude OR 4.98, 95% CI: 2.72–9.11, *p* < 0.001). Participants who had heard about cervical cancer had similar odds of screening uptake compared to those who had not (crude OR 0.60, 95% CI: 0.13–2.74, *p* = 0.511).

In multivariable logistic regression, adjusting for age and role (staff/student), older age remained independently associated with higher screening uptake, with each additional year of age increasing the odds by 11% (aOR 1.11, 95% CI: 1.05–1.18, *p* < 0.001). Similarly, being staff (compared to students) continued to be significantly associated with higher uptake (aOR 2.37, 95% CI: 1.15–4.92, *p* = 0.020). Knowledge and attitude were tested in the adjusted model but did not reach the *p* < 0.20 retention threshold and were therefore not retained in the final model (their crude ORs are nonetheless presented in Table 5 for completeness); religion and having heard about cervical cancer were likewise not retained. There was no important collinearity between age and staff/student status (variance inflation factors < 2), and the model showed adequate fit (Hosmer–Lemeshow χ^2^ = 6.42, df = 8, *p* = 0.60). The linearity of the association between age and the log‐odds of screening was assessed by adding age as a categorical (quartile) term and by a fractional‐polynomial check; neither indicated meaningful departure from linearity, so age was retained as a continuous linear term. Details in Table 5.

**Table 5:** Factors associated with uptake of Cervical Cancer screening among female students and staff of Gulu University.

Variable	Screening uptake		cOR (95% CI)	*p*‐value	aOR (95% CI)	*p*‐value
	No (%)	Yes (%)				
**Age,** median [IQR]	26 [23–27]	29 [27–29]	1.15 (1.10, 1.21)	< 0.001	1.11 (1.05, 1.18)	**< 0.001**
**Religion**						
Anglican	75 (32.61)	34 (32.38)	Reference			
Born Again	37 (16.09)	18 (17.14)	1.07 (0.54, 2.15)	0.842	N/A	
Catholic	99 (43.04)	47 (44.76)	1.05 (0.61, 1.79)	0.865		
Other	19 (8.26)	6 (5.71)	0.70 (0.26, 1.90)	0.48		
**Staff/Student**						
Student	209 (90.87)	70 (66.67)	Reference			
Staff	21 (9.13)	35 (33.33)	4.98 (2.72, 9.11)	< 0.001	2.37 (1.15, 4.92)	**0.020**
**Heard about cervical cancer**						
No	4 (1.74)	3 (2.86)	Reference			
Yes	226 (98.26)	102 (97.14)	0.60 (0.13, 2.74)	0.511	N/A	

*Note:* Age was analyzed as a continuous variable (in completed years); the values shown for age are the median and interquartile range within the screened and unscreened groups, not mutually exclusive age categories, so the ranges may overlap, and every participant, including those aged exactly 27 years, is included in one group only. Bold *p*‐values are the factors statistically significant in the final model. The odds ratios for age represent the change in the odds of ever having been screened for each additional year of age.

Abbreviations: aOR, adjusted odds ratio; CI, confidence interval; cOR, crude odds ratio; Ref, reference category.

## 4 Discussion

This study examined the knowledge, attitudes, uptake, and associated factors of cervical cancer screening among female students and staff aged 25–49 years at Gulu University, Northern Uganda. The findings revealed high general awareness of cervical cancer. The screening uptake in this study is higher than rates reported among university students in other parts of Uganda, such as 20.3% among female students at Kampala International University, Western Campus, Uganda [12]., and other parts of Africa, including 12.1% female students in Tanzania [30], 6.2% in South Africa [23], 14.4% in Kenya [24], 2.2% in Ethiopia [25] and 7.2% in Nigeria [26]. However, lower than 47.8% in Botswana [27], 47% in other parts of Tanzania [28], and 72.5% in Côte d'Ivoire [29].

The rate is also comparable to other population like 30.3% in a multi‐site Ugandan study involving women in HIV care [11] and 19.2% among HIV‐positive women in Ethiopia [31]. This result should be contextualized: the WHO 2030 elimination target of 70% coverage at ages 35 and 45 refers to lifetime screening within a program and is not directly comparable to cross‐sectional “ever screened” prevalence; we therefore present it only as a distal policy benchmark rather than a like‐for‐like comparison [1]. In a broader perspective, the uptake rate in this study exceeds pooled estimates of 12.87% in SSA [16], and 20.94% across the African region [32], as well as 11.6% from 25 low, middle‐income and emerging economy countries [33]. These comparisons suggest that while tertiary institutions in Northern Uganda may benefit from higher education levels facilitating moderate uptake, structural challenges in rural settings, like limited on‐campus screening services, contribute to suboptimal rates compared to urban or high‐resource contexts [20].

Knowledge levels in our cohort were generally good, aligning with 79% in Nigeria [34, 35]. This surpasses reports from similar populations. For instance, only 39% of university students in Western Uganda demonstrated good knowledge [12], 55% in Ethiopian undergraduates [21], and 19.7% in Kenya [24]. In sub‐Saharan Africa, pooled knowledge rates among women range from 40% to 60% [36], with gaps in HPV awareness mirroring our finding of 37% uncertainty about the causative agent, consistent with 35% in rural Ugandan women [19] and 42% in South African students [23]. Notably, knowledge in our cohort was higher than in a study of female university students in Saudi Arabia, in which 88.7% had unsatisfactory knowledge of cervical cancer; the two studies are therefore consistent in direction, with our participants comparatively better informed [35].

Attitudes toward screening were nearly evenly split, reflecting a moderate but insufficient level to drive high uptake. This is lower than 55% positive attitudes among Ethiopian university students [37] and 76% in in Kenya [38], 65% in urban Ugandan HIV‐positive women [14], and 76% in Kenya [38]. In sub‐Saharan Africa, negative attitudes often stem from stigma and low risk perception, as seen in 48% neutral or negative responses in Malawian studies [39]. This mixed attitude highlights actionable opportunities for behavioral change interventions, including peer‐led awareness campaigns, risk‐communication workshops, and integration of attitude‐shifting modules into university health education programs. Such strategies could help align perceptions more closely with existing knowledge, reduce stigma and fear‐based barriers, and ultimately bridge the gap between awareness and preventive action in this high‐potential population.

Beyond comparison with other studies, our findings point to a clear knowledge–action gap that warrants interpretation. Awareness of cervical cancer was almost universal (97.9%) and overall knowledge was high (83.3%), yet only 31.3% of women had ever been screened. This dissociation suggests that general awareness does not translate into screening behavior when specific, actionable knowledge is missing and when structural access is limited. Consistent with this, item‐level knowledge of the causative agent (HPV), of specific screening methods, and of risk factors was comparatively low, and the leading barrier to screening was simply not knowing where screening is offered. In other words, women knew that cervical cancer matters but frequently did not know how, where, or why to be screened, so high awareness coexisted with low uptake.

The higher uptake among staff compared with students may plausibly be explained by differences in autonomy, resources, and health‐system contact rather than differences in awareness; because these mechanisms were not directly measured, the following explanations are offered as hypotheses rather than established findings. Staff are, on average, older, financially independent, and more likely to have routine contact with health facilities (e.g., through employment‐based health checks or antenatal and postnatal care), all of which lower the practical and financial barriers to screening. Students, by contrast, are younger, more financially dependent, perceive themselves to be at low personal risk, and have fewer independent points of contact with reproductive‐health services. This interpretation is reinforced by the independent age effect observed in the adjusted model, in which each additional year of age was associated with higher odds of screening.

Institutional factors are central to explaining the low uptake in this population. The university is non‐residential and offers no on‐campus cervical cancer screening service, so students must actively seek screening at external facilities, incurring travel, cost, time away from study, and the discomfort of navigating an unfamiliar service. The absence of an on‐site, youth‐friendly screening pathway effectively converts high awareness into unmet demand. This suggests that the single most impactful institutional intervention would be to bring screening (or clear, low‐barrier referral and reminders) onto campus, paired with education that targets the specific knowledge gaps identified here. Interpreted this way, the results identify concrete, modifiable leverage points rather than simply documenting a prevalence figure.

Barriers identified in our study, particularly informational deficits on screening locations and fear of pain, align with regional patterns. In Uganda, similar barriers include embarrassment and rural inaccessibility [13], with pain fears reported by 30% of rural women [20]. Across sub‐Saharan Africa, financial constraints and stigma dominate, as per a systematic review [15], while perceived healthiness aligns with low risk perception in Ethiopian women [40]. Outside Africa, barriers like cost are less prominent in settings with universal healthcare, such as 15% in China [41], emphasizing resource disparities. These findings suggest multi‐level interventions, including mobile screening units in universities to address location barriers and counseling to mitigate fears, could enhance uptake.

Older age independently predicted higher screening, a finding consistent with studies across Africa. For example, in Ethiopia [37] and among rural Ugandan women aged 31–39 years [20]. This pattern likely reflects greater cumulative health literacy, increased perceived personal risk of cervical cancer, more frequent healthcare contacts, and fewer competing life priorities among older women compared with younger university students. The consistent age effect across diverse settings reinforces the robustness of this predictor and highlights the particular vulnerability of younger women in tertiary institutions, where lower risk perception and limited prior exposure to reproductive health services may contribute to delayed or absent screening.

Staff status also predicted uptake. We hypothesize, though did not directly measure, that this reflects better access to health services or higher socioeconomic status compared to students, aligning with employment as a facilitator in Ethiopian multicentre studies [40]. Similar findings have been reported among undergraduate students in Nigeria [42]. Staff members in tertiary institutions typically have greater financial autonomy, more stable access to healthcare information, regular exposure to workplace health programs, and fewer competing academic or social demands than students, all of which may lower practical and perceptual barriers to screening. In contrast, students, particularly younger undergraduates, often face competing priorities such as academic pressure, financial constraints, reliance on external funding, and lower perceived personal risk [43], contributing to delayed or absent screening behavior.

### 4.1 Strengths and Limitations of the Study

This study has several strengths that enhance its validity and relevance. First, the use of stratified random sampling across faculties and roles (students and staff) ensured a diverse and representative sample within the tertiary institution, capturing variations in academic backgrounds and experiences that could influence knowledge and attitudes toward cervical cancer screening. The high response rate minimizes non‐response bias and strengthens the reliability of the findings. Additionally, the questionnaire was adapted from a validated tool [21] and pilot‐tested, improving its cultural appropriateness and measurement accuracy.

Despite these strengths, the study has notable limitations. The cross‐sectional design captures associations at a single point in time, limiting the ability to establish causality between factors like knowledge, attitudes, and screening uptake. Longitudinal studies would be needed to track changes over time and infer causal relationships. Second, reliance on self‐reported data introduces potential biases, including recall bias for screening history, absence of objective verification through medical records (self‐report may overestimate actual uptake), and social desirability bias for sensitive topics like attitudes and barriers, which may lead to overestimation of knowledge or positive attitudes. Third, the study was conducted at a single tertiary institution in rural Northern Uganda, restricting generalizability to other universities, regions, or non‐academic populations with different socioeconomic or cultural contexts. For instance, the non‐residential nature of the institution and the focus on an educated cohort may not reflect barriers faced by less literate women in broader communities. Lastly, while the age range (25–49 years) targets high‐risk groups, it excludes younger students who are the majority at university and may be at emerging risk. Future research could mitigate these limitations by incorporating mixed‐methods approaches, objective verification of screening (e.g., medical records), and multi‐site designs for broader applicability.

Two further methodological considerations qualify the interpretation of our findings. First, the achieved sample (335 of a planned 336) was powered using a comparatively high awareness proportion (67.7%), which may have influenced the precision of some sub‐group estimates; the restricted 25–49‐year age bracket, chosen to reflect the age range at greatest cervical‐cancer risk and the group for whom routine screening is most strongly recommended, necessarily excludes the large population of younger undergraduates and limits generalizability to them. Second, the recruitment channels used (WhatsApp groups, class announcements, and direct contact) may have introduced self‐selection bias toward more health‐aware participants, which could have inflated the observed levels of awareness, knowledge, and, to a lesser extent, uptake. These factors should be borne in mind when interpreting the reported prevalence and associations.

## 5 Conclusion

Female students and staff at this tertiary institution in Northern Uganda exhibit high cervical cancer awareness and good overall knowledge, yet significant gaps persist in understanding HPV causation, symptoms, and screening methods. Attitudes remain mixed, and uptake is low at 31.3%; while not directly comparable, this remains far short of the WHO 2030 aspiration of 70% of women screened with a high‐performance test by ages 35 and 45 [1]. Key barriers, which included lack of information on screening locations, fear of pain, perceived good health, and shyness, highlight the persistent disconnect between knowledge, attitudes, and preventive action.

Older age and staff status independently predict higher uptake, indicating the influence of maturity, perceived risk, healthcare access, and institutional positioning on screening behavior. To address these findings, we recommend integrating comprehensive cervical cancer education into university curricula and orientation programs, establishing accessible on‐campus or mobile screening services, implementing peer‐led awareness campaigns to reduce fears and stigma, and prioritizing targeted outreach to the younger women within the studied 25–49‐year group, who showed the lowest uptake (noting that undergraduates below 25 years were not sampled and are a priority for future study). Universities represent strategic entry points for achieving national cervical cancer elimination goals. Institutionalizing on‐campus screening services and age‐appropriate risk communication could substantially improve uptake among the younger women in this age group, leverage the educated status of this population as agents of change in their communities, and contribute meaningfully to Uganda's cervical cancer prevention efforts and the global drive toward elimination by 2030.

These conclusions should be read in light of the study design and sampling. As a cross‐sectional study, it establishes associations rather than causation, so the age and staff‐status findings identify groups at higher or lower likelihood of screening rather than proven causal drivers. The restriction to women aged 25–49 years, chosen for its clinical relevance to peak cervical‐cancer risk and recommended screening ages, means these findings describe this specific group and should not be generalized to younger undergraduates, who form the majority of the student body and whose screening behavior warrants separate study. Accordingly, the recommendations above, integrating education into curricula, establishing on‐campus or mobile screening, and prioritizing outreach to younger students, are offered as implications and priorities for action and future research rather than as interventions already shown by this study to increase uptake.

## Author Contributions

**Milton Anguyo:** conceptualization, methodology, validation, investigation, writing – review and editing, project administration, supervision, resources. **Morrish Obol Okello:** conceptualization, methodology, software, data curation, investigation, validation, formal analysis, visualization, resources, writing – original draft, writing – review and editing. **David Komakec:** conceptualization, methodology, investigation, resources, writing – review and editing. **Emmanuel Alyoomu:** conceptualization, methodology, investigation, data curation, resources, writing – review and editing. **Habert Aziku:** conceptualization, investigation, methodology, data curation, visualization, resources, writing – review and editing. **Ruth Catherine Ouma:** methodology, data curation, visualization, writing – review and editing, formal analysis, validation. **Pebalo Francis Pebolo:** conceptualization, methodology, software, project administration, supervision, resources, writing – review and editing, visualization, validation, investigation, data curation.

## Funding

The authors have nothing to report.

## Use of Artificial Intelligence (AI)

As the authors are non‐native English speakers, the manuscript underwent language editing using Grammarly Premium software. This tool was used to improve grammar, spelling, punctuation, and sentence structure. All edits were manually verified by the authors to maintain the scientific integrity and accuracy of the content.

## Ethics Statement

Ethical approval was obtained from the Gulu University Research Ethics Committee (GUREC‐2023‐781). Written informed consent was secured, emphasizing voluntariness, confidentiality, and the right to withdraw. Data were anonymized and securely stored. No incentives were provided beyond study information.

## Conflicts of Interest

The authors declare no conflicts of interest.

## Data Integrity Statement

All authors have read and approved the final version of the manuscript. Morrish Obol Okello (corresponding author and manuscript guarantor) had full access to all of the data in this study and takes complete responsibility for the integrity of the data and the accuracy of the data analysis.

## Role of the Funding Source

This research received no specific external funding. No funding source or financial relationship had any involvement in the study design; collection, analysis, or interpretation of data; writing of the report; or the decision to submit the report for publication.

## Patient and Public Involvement

Patients or members of the public were not involved in the design, conduct, reporting, or dissemination plans of this research. The research had no funding or resources available to support meaningful public or patient involvement in the study process. The findings will be shared with the university community and relevant local health authorities to inform future awareness and screening initiatives.

## Transparency Statement

The manuscript guarantor (Morrish Obol Okello) affirms that this manuscript is an honest, accurate, and transparent account of the study being reported; that no important aspects of the study have been omitted; and that any discrepancies from the study as planned have been explained.

## Data Availability

The data that support the findings of this study are available on request from the corresponding author. The data are not publicly available due to privacy or ethical restrictions.
